# The Clinical Applications of Left Atrial Strain: A Comprehensive Review

**DOI:** 10.3390/medicina60050693

**Published:** 2024-04-24

**Authors:** Thomas O’Neill, Puneet Kang, Andreas Hagendorff, Bhupendar Tayal

**Affiliations:** 1 Department of Internal Medicine, University Hospitals Cleveland Medical Center, Cleveland, OH 44106, USA; 2 Department of Cardiology, Leipzig University Hospital, 04103 Leipzig, Germany; andreas.hagendorff@medizin.uni-leipzig.de; 3 Harrington Heart and Vascular Institute, University Hospitals Cleveland Medical Center, Cleveland, OH 44106, USA

**Keywords:** left atrial strain, left atrial function, strain imaging, LA strain

## Abstract

Left atrial (LA) strain imaging, which measures the deformation of the LA using speckle-tracing echocardiography (STE), has emerged recently as an exciting tool to help provide diagnostic and prognostic information for patients with a broad range of cardiovascular (CV) pathologies. Perhaps due to the LA’s relatively thin-walled architecture compared with the more muscular structure of the left ventricle (LV), functional changes in the left atrium often precede changes in the LV, making LA strain (LAS) an earlier marker for underlying pathology than many conventional echocardiographic parameters. LAS imaging is typically divided into three phases according to the stage of the cardiac cycle: reservoir strain, which is characterized by LA filling during systole; conduit strain, which describes LA deformation during passive LV filling; and booster strain, which provides information on the LA atrium during LA systole in late ventricular diastole. While additional large-population studies are still needed to further solidify the role of LAS in routine clinical practice, this review will discuss the current evidence of its use in different pathologies and explore the possibilities of its applications in the future.

## 1. Introduction

Speckle tracking echocardiography (STE) is an imaging technique that has been used to quantify and prognosticate cardiac functions since the early 2000s. The two-dimensional tracking of speckles, or acoustic markers of myocardium, is performed via automated software (EchoPAC software version203, GE Heathcare) and provides a functional assessment of myocardial contraction [1]. Initially, left ventricular (LV) global longitudinal strain (GLS) gained recognition in the early detection of LV systolic and diastolic function via STE [2]. Traditionally the focus of left atrial (LA) assessment has been limited to the assessment of the LA’s size using echocardiography and other advanced imaging modalities. LV function determined by LV filling is dependent on the LA mechanics of receiving and releasing blood to the LV. Recently, LA strain (LAS) assessment using STE has been found to provide a remarkable means to quantify phasic LA mechanics which has been recognized to be an even earlier marker of LV pathophysiology [3]. This review will summarize important clinical studies demonstrating the use of LAS in the present clinical landscape.

### 1.1. Assessment of LA Strain

The strain evaluation of the LA provides a unique assessment of the three phases of LA function based on the cardiac cycle. The reservoir phase describes the LA filling by the pulmonary veins during LV systole. This is the phase where the left atrium enlarges. The conduit phase describes the passive LV filling from the LA during early LV diastole. The booster-pump phase describes the atrial kick whereby the LA ejects blood as a form of active LV filling during late LV diastole. Both of these phases, conduit and booster, represent the shortening of the LA due to its emptying into the LV [3]. Each of these phases can be individually quantified using STE via echocardiography and each phase carries relevant information describing the integral functionality of the LA and LV.

While this review will focus on strain analysis using STE, it is worth noting the advanced imaging options which also provide a volumetric and functional analysis of the LA. Cardiac magnetic resonance (CMR) provides information on LAS using feature tracking, a technique similar to STE. Due to its accessibility and affordability relative to CMR, STE is most often the initial imaging modality used. Limited studies exist directly comparing the two methods; however, there appear to be slight variations in the measurements found depending on the modality used, as one study found that LAS was significantly lower with STE compared to CMR with a study population of 70 subjects (mean difference of 9.9 ± 12 for LASr) [4]. This study also found the reproducibility of these results to be higher with TTE (coefficient of variance 5.5–8.5% in STE vs. 9.9–17.9% in CMR). A third modality, cardiac computed tomography angiography (CCTA) is also available to evaluate LA morphology and function. A 2020 study comparing STE with CCTA found LASr to be significantly lower than CCTA, with a median LASr of 19.0 when using STE and 28.0 when using CCTA [5]. Although the spatial and temporal resolution may be higher in the advanced modalities, the reliability, accessibility, and relative affordability make STE an excellent first option for the evaluation of LA function and morphology.

**Figure 1:**
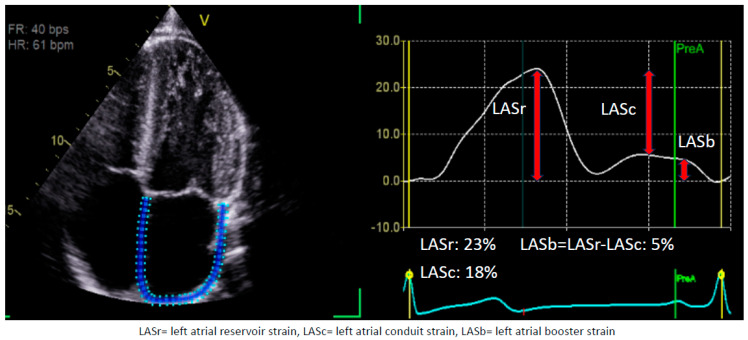
LA strain. This figure demonstrates the different components of left atrial strain. It is from a 28-year-old man. When tracing, it is important to exclude pulmonary veins’ ostia. As the figure highlights, we get all three phases of LA strain from a single tracing. This figure was obtained from the authors’ personal collection.

The measurement of each LA phase has been standardized as reported by a consensus document published by the European Association of Cardiovascular Imaging. The optimal window to assess LAS is an apical, non-foreshortened, four-chamber view (Figure 1). The reference time point would be the LV end-diastole (strain = zero) as determined by the mitral valve inflow. The automated software (EchoPAC software version203, GE Heathcare) requires users to define the atrial wall/endocardial border prior to performing calculations. Each phase of LAS is defined as follows: Reservoir stain is the peak strain before the opening of the mitral valve during the LV systole. Booster strain is the strain at the ventricular end-diastole minus the strain at the onset of atrial contraction. Conduit strain is defined as the difference between booster and reservoir strain [6]. This consensus allows for the standardization of LAS assessment via a semi-automated software (EchoPAC software version203, GE Heathcare) calculation which should, in theory, be reliable and reproducible.

### 1.2. Utility of LA Strain

Traditionally, volumetric analysis coupled with the transmitral flow’s peak velocities and velocity time integral were the predominant imaging parameters used to assess the phases of LA function. However, studies have shown that, especially in the case of LA reservoir strain (LASr), changes in LAS precede changes in LA volume [3]. Guan et al. found that the LAS rate was significantly different between different grades of diastolic dysfunction (DD), and they noted that this difference in strain rate was evident despite a lack of significantly different LA volumetric indices (LAVI) between the groups [7]. Several additional studies have suggested that LA dilation may lag changes in strain imaging by up to a decade as they relate to predicting the development of atrial cardiomyopathy [8,9]. While the precise pathophysiology of this remains incompletely understood, it is likely due in part to the atrial remodeling which occurs as a result of stress-induced fibrosis of the atria. This fibrosis results in functional changes in the atria prior to geometric changes [10,11,12,13]. One advantage of strain imaging is it is not as load or angle dependent as measurements of traditional volumetric indices via tissue Doppler imaging [14]. Some of the important clinical applications of LAS where research have shown a promising role are discussed in the following segments.

### 1.3. Hypertension and Diabetes Mellitus

LA morphologic and functional changes in patients with hypertension and diabetes mellitus are well described in the literature [15,16]. Specifically, these patients often have LA enlargement and increases in LA volume [17,18]. LA enlargement has wide-ranging clinical implications, including associations with an increased risk of stroke, heart failure (HF), and atrial arrhythmias [19]. For example, for each 5 mm increase in LA diameter, the risk of the development of atrial fibrillation (AF) may increase by as much as 39% [20], while every 10 mm increment in LA size was associated with a 140% relative increase in stroke risk [21]. A 2008 study even demonstrated that an increased LA volume predicts mortality independent of changes to the LV [22]. 

LAS is emerging as a sensitive tool to identify subclinical functional changes in patients with both hypertension and diabetes. Importantly, as several recent studies have demonstrated, these changes in LAS occur prior to the development of LA enlargement [23,24,25]. This suggests that LAS may serve as a more sensitive tool in identifying patients who are particularly at risk for the development of LA enlargement and its complications. This may also prove vital in selecting patients in whom more aggressive glucose and blood pressure control would be most beneficial. These findings, that LA function is impaired prior to identifiable geometric changes to the LA, perhaps provide evidence that an atrial myopathy precedes, and may contribute to, the LA enlargement which has been linked to stroke, HF, and arrhythmias [19]. These functional changes are thought to arise due to inflammatory and oxidative injury within the atria which results in interstitial fibrosis. Stefani et al. recently demonstrated that hypertension can induce such changes which result in an LA myopathy, as evidenced by reduced LASr and LA conduit strain, which precedes the development of LV hypertrophy or changes in the LA’s geometry. This study also found that patients with hypertension had a mean LASr of 29.78% (±6.08%) and an LA conduit strain of 14.23% (±4.59%) compared with 34.78% (±7.37%) and 19.66% (±7.29%) in non-hypertensive controls (*p*-value < 0.001) [26]. The effects of specific medications on LAS has also recently been investigated in patients with hypertension. Specifically, the use of spironolactone, demonstrated a trend towards improved LASr, although the difference did not reach statistical significance in a 2023 study from Girard et al. in patients with resistant hypertension over six months (29.1 ± 8.5% vs. 30.9 ± 5.5% *p*-value: 0.068) [27]. These hypothesis-generating studies demonstrate the need for further investigation into the effects medications may have in improving LAS in HTN. These data suggest that LAS may eventually be helpful in reducing cardiovascular (CV) morbidity and mortality in otherwise normal subjects with common health problems like hypertension and diabetes.

### 1.4. Chronic Kidney Disease

The association between chronic kidney disease (CKD) and cardiovascular disease (CVD) has been well established, with approximately half of all patients with CKD 4 or 5 having simultaneous CVD [28]. What is more, CVD significantly increases mortality for these patients, and is the cause of death for nearly 50% of patients with end-stage renal disease [29]. Geometric and morphologic changes have been noted in these patients with echocardiography and they typically have been reported to have left ventricular hypertrophy [30]. The typical changes seen on echocardiography include geometric changes to both ventricles, in addition to increased LA volume [31]. 

In addition to the morphologic changes seen, recent studies have demonstrated that functional changes in the LA occur in patients who have both advanced CKD and are on dialysis. Hassanin and colleagues, for example, found that LASr was decreased in patients on dialysis [32]. Furthermore, Gan et al. found in 2021 that lower LASr in CKD patients was associated with death from renal failure, a reduction in the estimated glomerular filtration rate to ≤15, or a doubling of serum creatinine [33]. Additionally, after three years, patients with LASr ≤ 23% developed the composite outcome in 20% of cases versus just 6% in those with LASr ≥ 23% [33]. Similarly, Gan and colleagues again found in 2021 that strain parameters could be useful in identifying patients with CKD who are at the highest risk for adverse cardiovascular outcomes [34]. After three years, patients with the largest reduction in LASr, defined as LASr ≤ 20.3%, experienced death or a major adverse cardiovascular event (MACE) in 42% of cases, compared with less than 10% for those with LASr ≥ 25%. These findings provide insight into the potential applications of LAS imaging in patients with CKD.

### 1.5. Atrial Fibrillation

AF is one of the most common arrhythmias, with an estimated prevalence of occurring in approximately 3% of adults worldwide [35]. The prevalence of AF is expected to rise, due in large part to an aging global population, increased rates of obesity, and increased detection [36]. Importantly, AF is associated with up to a two-fold increased risk of death, in addition to increased rates of stroke, cognitive impairment or dementia, myocardial infarction, sudden cardiac death, and HF [37]. While the pathophysiology of AF remains incompletely understood, mounting evidence suggests a complex interplay of inflammatory, fibrotic, and metabolic factors which eventually culminate in electrophysiologic volatility.

This growing global burden of AF necessitates early detection and effective, non-invasive strategies for monitoring and risk stratifying patients. In both of these areas, LAS has demonstrated exciting possibilities. A study from Hauser et al. in 2021 found that LAS independently predicted incidents of AF, even in patients without LA enlargement, and normal LV systolic function [38]. Decreasing LAS was also associated with an increased risk of AF over a mean follow-up period of 5.3 years. In this study, an LASr of 23% appeared to be an important threshold, as patients below this value were almost seven times more likely to develop AF than patients with an LASr greater than 23%. Additionally, the hazard ratio for the group with LASr < 15% was 22.14 (CI: 13.69–35.81) while the hazard ratio for patients with LASr between 19–23% was 4.16 (CI: 2.60–6.65). Crucially, reductions in LASr were observed across subgroup analyses, even prior to LA morphological changes. This is key, as many of the traditional echocardiographic parameters used in atrial fibrillation, such as LAVI, for example, have demonstrated efficacy in predicting AF recurrence following radiofrequency ablation [39]; however, this parameter is predicated on geometric changes of the LA. Hauser’s study demonstrated that functional changes, as measured by LAS, may anticipate the morphologic changes on which the traditionally used echocardiographic parameters are based. Additional studies are still required to directly compare the traditional parameters with LAS values to further determine the optimal studies to use for these patients. Furthermore, registry-based studies, as well as models leveraging artificial intelligence, have been used recently to identify EKG parameters that might predict atrial fibrillation development [40]. It will be of interest to see whether they are also associated with reductions in LAS or other morphological changes. This may eventually aid clinicians in risk stratifying patients according to their AF risk using LAS parameters, even before any other functional or structural changes are noted. 

In addition to the identification of at-risk patients with otherwise normal cardiac function, LAS values have also demonstrated the ability to identify high-risk patients in specific pathologic states. For example, in 2021 Jasic-Szpak et al. demonstrated that LAS indices, specifically LA booster strain and LASr, can help to predict the development of AF in patients with HF with preserved ejection fraction (HFpEF) [41]. In this study, LA booster strain, LASr, and LAVI were most predictive, with areas under the curve (AUCs) of 0.76, 0.71, and 0.72, respectively. As AF is frequently found in HFpEF, and its occurrence is associated with poor prognosis in these patients, as demonstrated by the meta-analysis from Mamas et al. in 2009 [42], the possibility of identifying those patients at a heightened risk of AF with LAS represents the potential for early and targeted monitoring and interventions for patients at a highest risk [42]. Similarly, LAS values were found to help predict the development of AF following an ST elevation myocardial infarction (STEMI) treated with percutaneous coronary intervention (PCI) [43]. For patients with diagnosed AF, LASr has also been found to outperform traditional risk scoring for identifying patients with an acute thrombus. Finally, in a 2014 study, Obokata et al. found that patients with acute left atrial appendage thrombus (LAAT) had a median LASr of 12.6% vs. 18.9% in controls with atrial fibrillation but no acute thrombus. Additionally, this study found that an LASr < 15.4% is a useful threshold, and had an AUC of 0.83 in identifying acute embolism, compared with the CHA2DS2-VASc score which had an AUC of 0.64 [44]. A 2023 study from Maheshwari et al. even found that LAS analysis may provide benefit in ischemic stroke risk stratification for patients with subclinical AF. The incorporation of LAS imaging with CHA2DS2-VASc in individuals without a history of stroke or AF enhanced stroke prediction [45].

**Figure 2:**
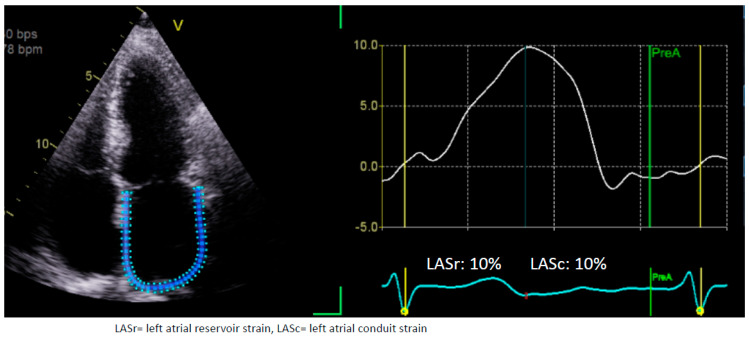
LA strain in atrial fibrillation. This is 62-year-old man with hypertension who came in with atrial fibrillation (AF). An attempt to direct cardioversion to sinus rhythm failed in this patient. With left atrial (LA) strain it can be noted that this patient has a severe LA myopathy with reduced LA reservoir strain. Note, there is no booster strain in this patient as there is no atrial kick due to AF. This figure was obtained from the authors’ personal collection.

Finally, LAS has demonstrated prognostic possibilities, in addition to diagnostic possibilities. A 2023 study demonstrated that higher LAS values were associated with the maintenance of sinus rhythm following catheter or surgical ablation [46]. LA booster strain values, in particular, were highly correlated with the maintenance of sinus rhythm, which likely reflects improved LA contractile function following ablation. LAS indices can be a surrogate marker for LA function, which may explain why patients with higher LAS values may be more likely to maintain their sinus rhythm [46]. The mean LASr was 22.6% (±8.5%) in the group that maintained their sinus rhythm following ablation at 3 months, while it was 16.7% (±5.7%) in the group in which AF recurred. Similarly, LA booster strain in the recurrence group was 5.6% (±2.5%) while it was 9.2% (±3.4%) in the sinus rhythm group. These findings further suggest the exciting potential of LAS indices’ use in the monitoring of patients with AF post ablation. An example of 63-year-old patients with AF who failed to cardiovert to sinus rhythm is shown in Figure 2 where the LASr is severely reduced. 

### 1.6. Heart Failure

HF is a complex clinical syndrome characterized by vascular congestion and volume overload in the setting of cardiac dysfunction. Recent estimates suggest that nearly 65 million people worldwide have HF [47]. Patients with HF are, broadly, categorized according to LV ejection fraction (EF), as patients with an EF ≥ 50% are characterized as having HFpEF, while those with an EF of ≤40% are diagnosed with HF with reduced EF (HFrEF). While treatments and clinical phenotypes differ between HFpEF and HFrEF, the five-year survival rates for both are approximately 50%, illustrating the need for improved diagnostic, therapeutic, and prognostic approaches [48]. LAS imaging has demonstrated exciting potential to provide additional clinically useful information in patients with HF. In fact, LAS imaging with both STE and CMR have demonstrated predictive capabilities in HF, as both modalities have identified changes in LAS prior to changes in the LV [49,50]. LAS has also shown association with lab findings indicative of vascular congestion. Pastore et al. demonstrated, in their 2023 study, that LASr is inversely related with the N-terminal-pro-brain-natriuretic peptide (NT-proBNP) in both acute and chronic HFpEF and HFrEF [51]. Their group also found that the combination of NT-proBNP with LASr may provide additional prognostic information as patients with both NT-proBNP values ≥ 875 pg/mL and LASr ≤ 15% had markedly reduced survival when compared with other subjects in the cohort. Additional studies will be required to further validate the usefulness of combining these two parameters for patients with both acute and chronic HF. Specific changes have been identified in the LAS parameters in patients with acute HF. Using a patient registry with over 4000 subjects, Park et al. found that LASr was a significant predictor of adverse events [52]. Their multivariable analysis further found that for each 1% increase in LASr, adverse clinical events decreased by 1.6% in acute HF. Of note, these findings were consistent across subjects with HFrEF, HFmrEF, and HFpEF. Although additional research is required to validate these results, they suggest that LAS parameters could potentially assist in the prognosis of patients with acute HF.

### 1.7. Heart Failure with Preserved Ejection Fraction (HFpEF)

LA dysfunction is increasingly recognized as a key component in the pathophysiology of HFpEF [53,54,55]. Recently, LAS has shown the potential to play both a crucial diagnostic role, as well as prognostic role in this syndrome. In 2019, Reddy et al. demonstrated that LASr consistently outperformed traditional echocardiographic parameters used in the diagnosis of HFpEF, including the E/e’ ratio, LV GLS, and LV hypertrophy [56]. In this study, HFpEF patients were identified based on their clinical symptoms, having an EF of >50%, and elevated cardiac filling pressures through invasive measurement techniques. Of the strain indices, LASr was found to have the highest diagnostic ability (AUC 0.719, *p*-value < 0.0001). Importantly, LASr alone nearly equaled the performance of a consensus recommendation tool developed by the European Society of Cardiology in 2019 which incorporates multiple conventional echocardiographic indices, including septal and lateral annular peak early diastolic velocities, the tricuspid regurgitation velocity, LA size, and the LV mass index [57]. This suggests that a single index can help in identifying elevated filling pressures rather than a complex multi-parametric approach. However, further data is required to support these initial promising findings. A recent meta-analysis provided more robust evidence which included 1906 patients from 20 studies and demonstrated that LASr had greater sensitivity in diagnosing elevated LV filling pressures than both E/e’ and LAVI, which are two of the most commonly used parameters to diagnosis HFpEF [58]. 

Obesity, a comorbidity commonly found in HFpEF patients [59], has also been associated with changes in LAS. Even in patients without known CVD, Aga et al. found, in 2023, that obese patients had a significant reduction in LAS parameters compared with non-obese controls [60]. Specifically, their group found their LASr to be significantly lower compared that of to controls (32.2 ± 8.8% vs. 39.6 ± 10.8% *p*-value: <0.001). This suggests that functional LA changes may occur prior to development of CVD. Further investigations will be needed to determine whether LAS parameters may help in predicting patients who may develop HFpEF.

In addition to assisting in making the diagnosis, LAS has also demonstrated clinical utility in assessing and quantifying the degree of DD seen in patients with HFpEF. As Singh and colleagues demonstrated in their 2017 study, unlike the other measures often used to assess DD, LAS progressively worsens depending on the severity of DD, suggesting strain parameters might have additional clinical utility in assessing and stratifying disease severity [61]. They found that an LAS threshold of 19% was 95% accurate in diagnosing patients with grade 3 DD. They further found that although a more traditional parameter, LAVI, also increased with the progressive worsening of DD, LAVI was not able to discriminate between grade 2 or grade 3 DD. In this particular study, the 2009 guidelines were applied to define DD grades. Grade 2, or moderate dysfunction, according to the 2009 guidelines, can be defined as the mitral E/A ratio of 0.8–1.5, and decreases by ≥50% during the Valsalva maneuver, the E/e’ ratio is 9–12, and e’ is <8 cm/s. Grade III dysfunction is a severe form in which restrictive LV filling occurs with an E/A ratio ≥ 2, a deceleration time < 160 ms, IVRT ≤ 60 ms, a systolic filling fraction ≤ 40%, and an average E/e’ ratio > 13 [62]. Moreover, LAS indices also provide valuable prognostic information in HFpEF, as Inciardi et al. found in 2022 that reduced LASr was associated with worse outcomes, even after correcting for both LV hypertrophy, or abnormal LV GLS [63]. 

### 1.8. Heart Failure with Mildly Reduced Ejection Fraction (HFmrEF)

While the use of LAS imaging has grown in both HFrEF and HFpEF, little is still known about its potential clinical utility in HF with mildly reduced EF (HFmrEF). HFmrEF, relatively recently recognized as a distinct clinical entity, is thought to account for 10–25% of HF diagnoses [64]. It is characterized by features which overlap both HFrEF and HFpEF syndromes simultaneously. A 2019 study from Al Saikhan and colleagues demonstrated notable changes in LAS for HFmrEF patients. Specifically, their group compared patients with HFmrEF to those with HFpEF. They found that patients with HFmrEF had worse LASr and LA booster strain than those with HFpEF [65]. Interestingly, these differences in LAS were observed between the two groups despite minimal differences in conventional echocardiographic parameters like LA size. The study provides insight into the functional changes in HFmrEF and illustrates the need for additional investigations into this phenotype.

### 1.9. Heart Failure with Reduced Ejection Fraction (HFrEF)

Although patients with HFrEF generally have a poorer prognosis than those with HFpEF just because of poor LV systolic function, LAS can be utilized to further categorize these patients to identify patients at an especially high risk. For example, a study from 2019 found that patients with lower LASr were more likely to have a recurrence of HF symptoms following the acute treatment of vascular congestion [66]. While the baseline LASr in this study was not predictive of mortality, less recovery of LASr following decongestive therapy was associated with worse outcomes. On the other hand, conventional parameters like E/e’ or LAVI were not predictive, a finding which suggests the potential role of LAS indices in identifying patients with a high-risk phenotype, or more advanced disease. Additionally, there has been growing evidence that LAS indices may help to predict the patients with HFrEF whose EF will improve. Torii and colleagues found in 2019 that higher LAS is associated with improvement in LV function in patients with reduced ejection fraction [67]. In this study, patients with an improvement in their EF to it being >40% within the 6-month follow-up period had a mean LASr of 13.0% (±1.5%) while those patients whose EF did not recover had a mean LASr of 10.1% (±1.1%). Of note, there was no significant difference between the two groups in other conventional echocardiographic parameters like E/e’ ratio or LAVI. This represents an important finding, and suggests that measuring LAS parameters during follow up for HFrEF patients may be of help in guiding management. While HFrEF can be caused by a diverse range of pathologies, investigations of LAS parameters in specific disease states are underway to better understand how LA function may contribute to HFrEF. LV noncompaction syndrome (LVNC), a genetic cardiomyopathy often resulting in reduced systolic function [68], was investigated in 2014 by Nemes et al. This group found that patients with LVNC had significantly lower LASr compared with control subjects (12.8 ± 8.2% vs. 22.5 ± 8.5% *p*-value: 0.004) [69]. Additional investigations are needed to further clarify these findings, as well as how LA function may change in other cardiomyopathies. Additionally, Carluccio et al. found in 2018 that LAS may predict adverse outcomes, including mortality, in HFrEF, independent of other clinical and echocardiographic measures; patients with an LASr of less than 12.9% were found to have lower rates of survival at 2.5 years compared with patients with a higher LASr [70]. Finally, as medical management with guideline directed medical therapy (GDMT) is a cornerstone of treatment for HFrEF, investigations are underway to determine whether these agents cause changes in LAS parameters. While evidence at this time is somewhat limited, Suo et al. recently demonstrated that the use of sacubitril/valsartan in animal models was associated with improved LASr in a retrospective study of patients with atrial fibrillation [71]. This study demonstrates the need for additional investigations into how medical therapies in HF may change LAS parameters over time.

## 2. Valvular Heart Disease

The challenge of determining the optimal timing for valvular intervention has been a long-standing focus of clinical investigation. The incorporation of GLS into the planning of such an intervention has garnered interest due to its improved sensitivity in the early prediction of mortality and symptom progression even in patients with preserved EF [2]. The characterization of functional abnormalities preceding tangible, structural changes carries prognostic value among patients who would not traditionally receive valvular intervention (asymptomatic severe aortic stenosis). 

### 2.1. Mitral Valve Disease

More recently, LAS has emerged as a prognostic tool for patients with left-sided valvular diseases. LAS has garnered even more interest in perioperative planning for severe mitral regurgitation (MR). Traditionally, LV GLS has a proven utility for the early detection and risk stratification of patients with asymptomatic MR, both moderate and severe. Mandoli et al. hypothesized that LAS provides additional sensitivity in detecting indications for mitral valve surgery that likely precede derangements in GLS. The underlying theory is that the LA is thin-walled and less likely to accommodate volume or pressure overload when compared to the thick-walled, muscular LV. As a result, the LA is hypothesized to be the earliest chamber to exhibit subclinical dysfunction with a decline in LAS due to atrial fibrosis in severe MR. Among a cohort of 71 patients with primary severe MR, Madoli et al. found an LASr < 21% to be a useful threshold when risk-stratifying patients, with those patients with an LASr < 21% having significantly lower 5-year event-free survival when compared to patients with an LASr ≥ 21%. Furthermore, this cohort exhibited normal LV GLS, supporting the notion that changes in LAS arise earlier than changes in LV strain in response to volume overload with severe MR [72].

Moreover, LAS may play a role in the prognosis of patients undergoing mitral valve intervention. Patients undergoing mitral valve repair (MVR) have been found to have reduced LV GLS and LASr post-procedurally, which represents the unmasking of the LA and LV dysfunction which likely stems from chronic, maladaptive remodeling in the two chambers from chronic volume overload [73]. Furthermore, subclinical atrial remodeling and fibrosis from chronic volume overload, represented by impaired LAS, has been linked to the development of postoperative AF [74,75]. 

Another population of interest is the cohort of asymptomatic, moderate MR. LAS is a sensitive predictor of an MACE in such patients. Cameli et al. studied a cohort of 276 patients with asymptomatic MR and found that reduced LASr and increased LAVI were the strongest predictors of CV events. With receiver operating characteristics (ROCs) curve analysis, an LASr of  <35% was found to be the optimal cutoff value (AUC LASr: 0.87). These findings demonstrate the possible utility and superiority of LAS over both GLS and LAVI in the risk stratification and application of strain as a parameter to carefully and closely follow these patients [76]. 

### 2.2. Aortic Stenosis

Similar to the challenges in identifying the optimal timing for mitral valve intervention, there is substantial emphasis on optimizing the risk stratification of patients with aortic stenosis (AS). This is important because the LV is prone to fibrosis in response to chronic pressure overload from AS. The prediction of the progression toward HF as well as identifying patients who would benefit from valvular intervention is of particular interest. LV GLS has been well-studied as a prognostic indicator of CV outcomes following aortic valve (AV) replacement with a higher sensitivity than traditional parameters (transvalvular gradients, AV area, symptom development) [2]. More recently, LAS has been identified as an even earlier predictor of CV outcomes in AS. 

**Figure 3:**
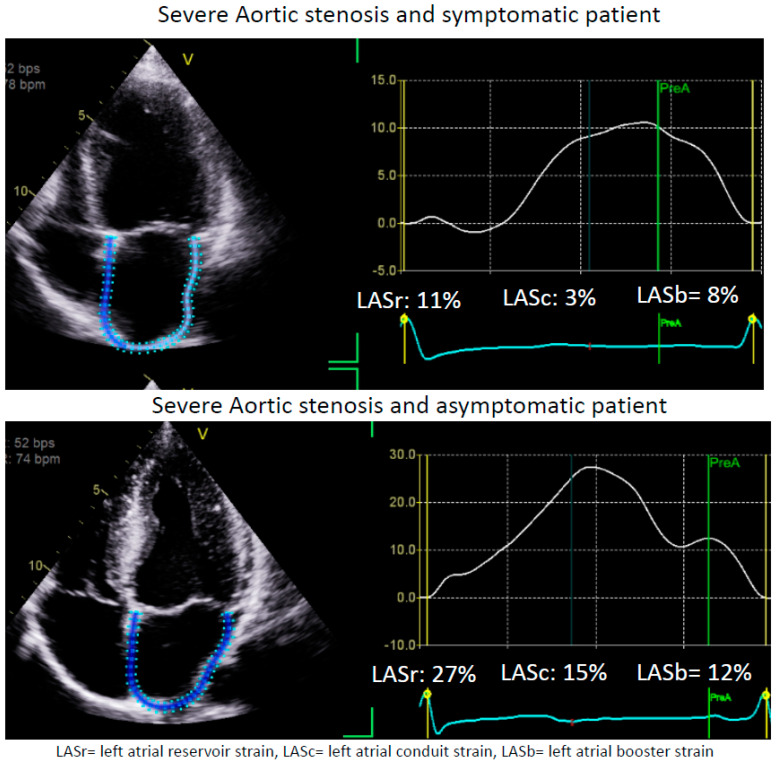
LA strain in aortic stenosis. These are two example cases of patients with severe aortic stenosis (AS). The upper panel is of a 62-year-old patient who is severely symptomatic, while the lower panel is of a 46-year-old patient with asymptomatic AS with a bicuspid aortic valve. Interestingly, the left atrial strain (LAS) is severely reduced in the patient in the upper panel, directly correlating with his symptoms. On the other hand, it is preserved in the patient in the lower panel despite this patient having secondary changes due to AS with severe left ventricular (LV) hypertrophy. Of note, the ejection fraction (EF) for the patient in the upper panel is 60–65%, with an LV mass of 118 g/m^2^, while the EF for the patient in the lower panel is 65–70% with an LV mass of 135 g/m^2^. This figure was obtained from the authors’ personal collection.

Specifically, LAS may offer unique utility in cases of asymptomatic moderate AS, since moderate AS is associated with poor prognosis [77]. Sonaglioni et al. studied a cohort of asymptomatic, moderate AS and followed them for CV hospitalization (CHF, ACS, arrhythmias), cardiac or sudden cardiac death, and found that preserved LASr was independently associated with a 15% lower risk of the composite outcome beyond the traditional risk factors [78]. LAS > 19% was identified as the cutoff with optimal sensitivity/specificity to predict CV events [78]. This finding highlights the importance of LAS in the risk stratification and procedural planning for a cohort of patients who were not traditionally identified as candidates for valvular intervention. Two example cases of severe AS with differential presentations are shown with Figure 3. 

Similarly, decreases in LASr after transcatheter aortic valve replacement (TAVR) may hold prognostic value relating to the prediction of MACEs. In a single-center observational study, of patients with severe symptomatic AS who underwent TAVR, it was found that the improvement in delta LAS post-TAVR was a strong, positive predictor of HF hospitalization and/or CV mortality (HR = 0.76; CI 0.67–0.86). In fact, an improvement in LASr was the only independent predictor of the combined clinical endpoint [79]. This finding signifies the utility in incorporating LAS in identifying post-TAVR patients who are more likely to have adverse outcomes, but further larger studies would be necessary to substantiate these findings.

### 2.3. Hypertrophic Cardiomyopathy

Hypertrophic cardiomyopathy (HCM) is a heritable cardiomyopathy characterized by heterogeneous myocardial hypertrophy and dynamic LV outflow tract (LVOT) obstruction. The incorporation of strain imaging in the diagnosis and prognostication of HCM has shown promise given the ability to identify functional changes that precede structural adaptations to altered hemodynamics due to obstruction and impaired sub-clinical LV function. LV GLS has been well-studied to correlate with adverse outcomes in HCM patients [80]. LAS carries prognostic value in HCM for many of the same reasons seen with the pathologies discussed above, owing to the early functional adaptations that are seen due to the thin-walled LA reacting to stress earlier than the thicker, adaptable LV. In addition, LAS has the unique utility of predicting the development of AF in patients with HCM which is known to be associated with increased adverse outcomes among HCM patients [81]. Hussain et al. conducted a systematic review and meta-analysis to study HCM patients and the utility of LAS in predicting the development of AF as well as MACEs. They found that all three LAS indices were associated with development of new AF in HCM. Additionally, it was found that all three types of LAS were significantly associated with an increased number of MACEs [82,83]. 

Recently, Saijo et al. studied LAS (reservoir, conduit, and booster) as a predictor of worsening exercise tolerance and the requirement for septal reduction therapies. They found that, across all quartiles of exercise tolerance, LAS worsened independent of traditional LAVI. They were also able to identify a cut-off for the LA booster strain of −13.9% for identifying exercise intolerance (AUC 0.68; sensitivity 74%, specificity 57%; *p* < 0.001). This association with booster strain has been well-studied as a compensatory mechanism to maintain cardiac output by increasing LV filling during exercise. A reduction in booster strain, therefore, would be expected to predict exercise intolerance in the general population and especially in patients with HCM [80]. Further large-scale, prospective investigation is necessary to determine how this finding may incorporate LAS in procedural planning among patients with HCM. 

### 2.4. Cardiac Amyloidosis

Cardiac amyloidosis (CA) is a form of restrictive cardiomyopathy characterized by an intra-myocardial deposition of disorganized amyloid fibril precipitating a thickened ventricular wall with DD [84]. The differentiation of this phenotype of restrictive cardiomyopathy from other forms of HCM has presented a diagnostic dilemma with important implications for management and prognosis.

**Figure 4:**
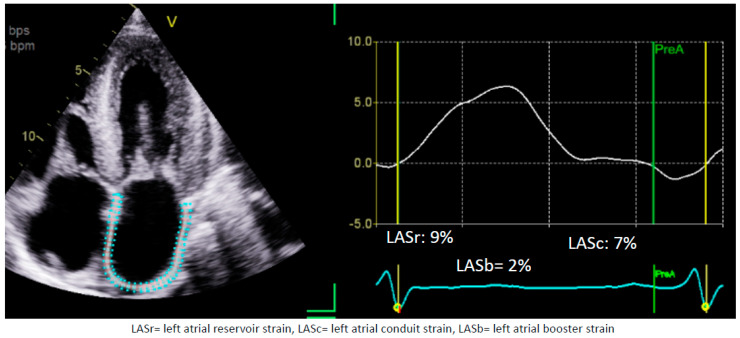
LA strain in cardiac amyloidosis. This is an example case of a 72-year-old patient with cardiac amyloidosis who has severe heart failure symptoms. The left atrial strain is severely reduced in this patient despite the LA not showing signs of severe dilatation yet. The left atrial volume index (LAVI) for this patient is 41 mL/m^2^. This figure was obtained from the authors’ personal collection.

The incorporation of strain imaging via STE has attracted attention in differentiating hypertensive heart disease from CA. With regard to GLS, the characteristic regional strain pattern associated with CA has been well-published (with impaired strain at basal segments and preserved strain in apical segments) [2]. More recently, LAS has been hypothesized as an even earlier marker for otherwise subclinical LV dysfunction and remodeling (Figure 4) [84]. Oike et al. performed a retrospective cohort study on patients with ATTR amyloidosis over a 2-year follow-up. They identified preserved LASr to be a clinically significant predictor of lower CV death among the cohort (OR 0.84; 95% CI 0.72–0.98; *p* < 0.05), while the traditional LV parameters (including LVEF, E/e’ ratio, and GLS) were not [85]. 

LAS has also shown utility in the prediction of outcomes in CA. Akintoye et al. performed a retrospective cohort study on patients with either AL or ATTR CA and no history of AF to study the prognostic utility of LAS in predicting thrombotic events, new AF, and mortality. Over a median follow-up of 3.8 years, they identified reduced LASr as not only being associated with higher mortality but also additional events including the onset of AF and thrombotic events. However, they noted that the strength of LASr as a predictor of mortality was greatest in the ATTR subset in comparison to the AL subset. Moreover, the incremental addition of LAS to traditional prognostic staging systems improved the sensitivity of the staging system [86]. These findings help to illustrate the possibilities of LAS to not only provide prognostic data for patients with CA but also for a comprehensive follow-up measure for other, softer end-points in these patients. 

### 2.5. Future Directions

**Figure 5:**
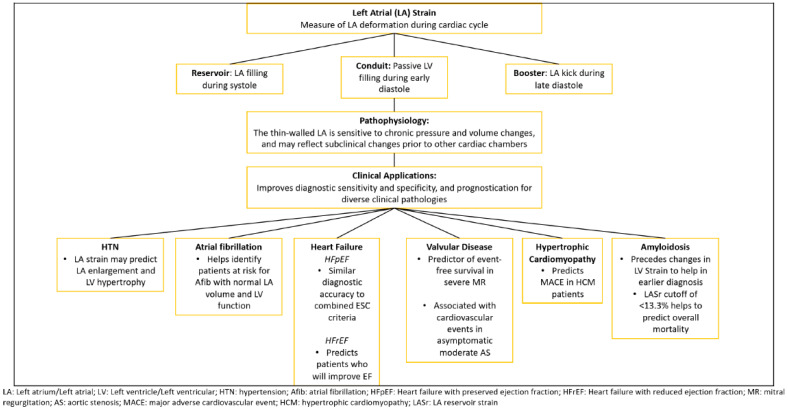
Central illustration. Summary figure describing the basic types, pathophysiology, and clinical applications of left atrial strain.

While LAS has yet to be integrated into routine clinical practice, mounting evidence, particularly within the past decade, demonstrates its utility in diverse clinical scenarios (Figure 5, Table 1 and Table 2). In addition to the use of LAS in the clinical syndromes described above, future investigations may demonstrate its use in other clinical entities. For example, although evidence at this time is somewhat limited, there may eventually be a role for LAS imaging in coronary artery disease, as Li et al. found that LAS could provide prognostic information for patients with acute coronary syndrome, as lower LASr was associated with increased rates of MACEs following acute coronary syndrome [87]. Across multiple cardiac diseases including valvular heart disease, HF, and AF, LAS imaging has demonstrated exciting potential to assist in diagnosis, categorization, and prognosis. The recent use of machine learning models that incorporate LAS to diagnose and risk stratify patients with DD has also shown promise, and may eventually allow for the incorporation of strain measurements into a multi-parametric approach [88,89]. While the use of machine learning and artificial intelligence in LAS imaging is still in its early stages, there is clear potential to leverage these technologies to identify subtle differences among a range of pathologies. It is possible that the incorporation of LAS may make the quantification of DD simpler than what is recommended currently. Large-scale studies are still needed, however, to verify and standardize some of the findings discussed in this review. The further clarification and specification of techniques and reference ranges, for example, is required to make LAS a useful modality in routine clinical practice. Additionally, investments in the education of technicians and sonographers will be vital to ensure the images obtained are of appropriate quality. Continued technological advancements may also assist in transitioning LAS imaging from a novel imaging tool used in research studies to an invaluable technique in providing care for some of the most clinically complex patients.

## 3. Conclusions

LAS has demonstrated potential in the diagnostic and prognostic assessments of patients with diverse pathologies. Additional studies are still required to formalize its use in routine clinical practice, but it may eventually provide invaluable insights for providers with minimal downside for patients.

## Figures and Tables

**Table 1:** Recent studies investigating the utility of left atrial strain in hypertension and diabetes, atrial fibrillation, and heart failure (with preserved and reduced ejection fraction).

Year	Author	Type of Study	No. of Subjects	Major Findings
**Hypertension and Diabetes Mellitus**				
2011	Mondillo et al. [23]	Single-center, prospective case–control	155	LAS is decreased in patients with HTN and diabetes
2021	Zhao et al. [25]	Single-center, prospective case–control	276	Decreases in LAS are found in patients with HTN prior to LA or LV enlargement
2015	Xu et al. [24]	Single-center, prospective case–control	248	LAS imaging identifies functional changes before morphologic changes in patients with HTN
2023	Stefani et al. [26]	Retrospective, cross-sectional	208	LAS imaging provides evidence of LA myopathy in patients with HTN
**Chronic Kidney Disease**				
2016	Hassanin et al. [32]	Prospective, single-center case–control	140	LASr reduced before LA dilation in dialysis patients
2016	Calleja et al. [90]	Retrospective, single-center cohort study	159	Decreased LASr seen in ESRD patients
2021	Gan et al. [34]	Multicenter, prospective cohort study	243	LAS helps to predict adverse cardiovascular outcomes in CKD
2021	Gan et al. [33]	Multicenter, prospective cohort study	280	LAS may predict ESRD in patients with CKD
2016	Unger et al. [91]	Prospective, single-center cohort study	299	CKD was associated with lower LASr
**Atrial Fibrillation**				
2021	Jasic-Szpak et al. [41]	Single-center, prospective cohort study	170	LAS can predict AF incident in HFpEF
2021	Hauser et al. [38]	Multicenter, prospective cohort study	4466	LAS predicts the development of AF in patients with normal LA volume and LVEF
2023	Khan et al. [46]	Substudy of randomized control trial (CASA-AF)	83	Improved LAS parameters were associated with the maintenance of SR following ablation
2022	Svartstein et al. [43]	Single-center, prospective cohort study	392	LAS values help to predict AF following STEMI treated with PCI
2014	Obokata et al. [44]	Multicenter, Prospective Observational Study	93	LASr outperformed CHA_2_DS_2_-VASc in identifying AF patients with an acute thrombus
**Heart Failure with Preserved Ejection Fraction (HFpEF)**				
2019	Reddy et al. [56]	Single-Center Observational	238	LASr offers more improved diagnostic information than many conventional parameters
2019	Telles et al. [92]	Single-Center Observational	71	Impaired LAS is associated with abnormal exercise hemodynamics in HFpEF, a marker for symptoms and survival in HFpEF
2022	Inciardi et al. [63]	Multi-Center Observational	4901	LAS outperforms LA volume in identifying patients at risk for HF
2022	Dal Canto et al. [58]	Systemic Review and Meta-analysis	1906	Normal LAS can essentially rule out HFpEF
2017	Singh et al. [61]	Retrospective cohort study	229	Worsening LAS can accurately discriminate between gradations of DD
2014	Santos et al. [93]	Retrospective, multicenter cohort study	135	There was an increased incidence of AF and HF hospitalization with lower LAS
2016	Santos et al. [94]	Retrospective, multicenter cohort study	357	LAS was associated with increased hospitalization and death
**Heart Failure with Reduced Ejection Fraction (HFrEF)**				
2016	Sanchis et al. [95]	Prospective, single-center cohort study	154	LAS may provide prognostic information, identifying patients at a higher risk
2019	Deferm et al. [66]	Prospective, single-center cohort study	31	Lower LASr was associated with the recurrence of decompensated HF
2020	Castrichini et al. [96]	Prospective, single-center cohort study	77	LASr was a surrogate measure for reverse remodeling in HFrEF patients treated with sacubitril/valsartan
2021	Torii et al. [67]	Prospective, single-center cohort study	100	LAS may predict which patients with HFrEF improve their EF
2018	Carluccio et al. [70]	Prospective, single-center cohort study	405	LASr provides prognostic information in HFrEF independent of LV GLS

LAS: left atrial strain; HTN: hypertension; LA: left atrial; LASb: left atrial booster strain; LV: left ventricular; AF: atrial fibrillation; LASr: left atrial reservoir strain; SR: sinus rhythm; STEMI: ST-elevation myocardial infarction; PCI: percutaneous coronary intervention; HFpEF: heart failure with preserved ejection fraction; DD: diastolic dysfunction; HFrEF: heart failure with reduced ejection fraction; GLS: global longitudinal strain.

**Table 2:** Recent studies investigating the utility of left atrial strain in valvular disease, hypertrophic cardiomyopathy, and cardiac amyloidosis.

Year	Author	Type of Study	No. of Subjects	Major Findings
**Mitral Valve Disease**				
2022	Samrat et al. [97]	Prospective, single-center observational	260	LAS improvement following MBV was a sensitive, early indicator of the maintenance of SR when compared to traditional LAV
2021	Mandoli et al. [72]	Prospective, single-center observational	65	LAS is an independent predictor of event-free survival in primary severe MR
2016	Toprak et al. [98]	Prospective, single-center cohort	25	LAS improved following TEER; Pre-operative LAS was associated with MACEs at the 1-yr follow-up
2019	Cameli et al. [76]	Prospective, single-center cohort	235	LAS was associated with cardiovascular events in patients with moderate MR
**Aortic Stenosis**				
2021	Sonaglioni et al. [78]	Retrospective, single-center cohort	186	LAS was independently associated with cardiovascular events in patients with asymptomatic, moderate AS
2021	Sabatino et al. [79]	Retrospective, single-center case–control	100	Changes in LAS following TAVR independently predicted HFH and cardiovascular mortality
**Hypertrophic Cardiomyopathy**				
2024	Hussain et al. [82]	Systematic review and meta-analysis	30 studies	All 3 LAS phases were predictors of new AF and MACEs in HCM patients
2022	Saijo et al. [99]	Prospective, single-center cohort	532	LAS correlates with reduced exercise capacity and diastolic dysfunction in HCM patients
2021	Tayal et al. [83]	Retrospective cohort study	50	LASr and LASc were associated with exercise tolerance and all 3 phasic LASs were associated with atrial fibrillation
**Cardiac Amyloidosis**				
2021	Oike et al. [85]	Retrospective, single-center cohort	113	LAS was an early predictor of cardiovascular death in ATTR CA patients; an LASr cutoff of <6.69% predicted cardiovascular death and HFH
2023	Akintoye et al. [86]	Prospective, registry-based cohort	448	LAS was an independent predictor of TE, new AF, and mortality in CA patients; an LASr cutoff of <13.3% predicted overall mortality
2020	Rausch et al. [100]	Retrospective, single-center cohort	79	LAS was significantly reduced in CA compared to HT groups; an LASr cutoff of <20% differentiated CA from HT cardiomyopathy

MBV: mitral balloon valvuloplasty; LAV: left atrial volume; MR: mitral regurgitation; TEER: transcatheter edge-to-edge repair; MACE: major adverse cardiovascular event; HFH: heart failure hospitalization; ACS: acute coronary syndrome; TAVR: transcatheter aortic valve replacement; SAVR: surgical aortic valve replacement; HCM: hypertrophic cardiomyopathy; LASc: left atrial conduit strain; ATTR CA: transthyretin cardiac amyloidosis; TE: thrombotic event; CA: cardiac amyloidosis; HT: hypertensive.
